# Visual Intuitions in the Absence of Visual Experience: The Role of Direct Experience in Concreteness and Imageability Judgements

**DOI:** 10.5334/joc.328

**Published:** 2024-01-09

**Authors:** Marco A. Petilli, Marco Marelli

**Affiliations:** 1 University of Milano–Bicocca, Milan, Italy; 2 NeuroMI, Milan Center for Neuroscience, Milan, Italy

**Keywords:** Visual experience, Concreteness, Imageability, Grounded cognition, Perceptual ratings, Blindness

## Abstract

The strongest formulations of grounded cognition assume that perceptual intuitions about concepts involve the re-activation of sensorimotor experience we have made with their referents in the world. Within this framework, concreteness and imageability ratings are indeed of crucial importance by operationalising the amount of perceptual interaction we have made with objects. Here we tested such an assumption by asking whether visual intuitions about concepts are provided accurately even when direct visual experience is absent. To this aim, we considered concreteness and imageability intuitions in blind people and tested whether these judgments are predicted by Image-based Frequency (IF, i.e. a data-driven estimate approximating the availability of the word referent in the visual environment). Results indicated that IF predicts perceptual intuitions with a larger extent in sighted compared to blind individuals, thus suggesting a role of direct experience in shaping our judgements. However, the effect of IF was significant not only in sighted but also in blind individuals. This indicates that having direct visual experience with objects does not play a critical role in making them concrete and imageable in a person’s intuitions: people do not need visual experience to develop intuition about the availability of things in the external visual environment and use this intuition to inform concreteness/imageability judgments. Our findings fit closely the idea that perceptual judgments are the outcome of introspection/abstraction tasks invoking high-level conceptual knowledge that is not necessarily acquired via direct perceptual experience.

## Introduction

At an operational level, the concreteness and imageability of a word referent are typically obtained through explicit human intuitions. Indeed, concreteness is measured by explicitly asking participants to rate how concrete (vs abstract) a word is, while imageability ratings are obtained by asking how easy it is to form a mental image of the word referent (Altarriba et al., 1999; Paivio et al., 1968; Toglia & Battig, 1978). Although imageability seems to be more visually biased than concreteness, these measures are highly correlated (Connell & Lynott, 2012; Speed & Brybaert, 2022; Vergallito et al., 2020) and often used interchangeably (Binder et al., 2005; Fliessbach et al., 2006).

What exactly makes a word referent concrete and imageable? The strongest formulations of grounded cognition (Barsalou, 1999, 2008; Meteyard et al., 2012) propose that our perceptual states acquired during the interaction with objects in the world make them concrete and imageable. Imageability and concreteness can thus be defined by the richness of sensory – primarily visual^1^ – information that a person can reenact from past experiences. According to this proposal, perceptual intuitions can “*provide a reasonable proxy for direct sensorimotor experience*” (Wingfield & Connell, 2022) by directly operationalising the amount of perceptual interaction we have in our everyday experience with objects (Connell & Lynott, 2012, 2014; Speed & Brybaert, 2022). Thus, the metrics of concreteness and imageability are, in principle, precious tools to study perceptual processes involved in cognition, being considered purely embodied or grounded measures, crucially linked to direct interactions with the world. In terms of the classic semiotic triangle (Cherry, 1957; Ogden & Richards, 1923) – which describes how symbols (i.e., words) relate to references (i.e., the concept representations) and referents (i.e., the objects) – grounded cognition considers concreteness and imageability as properties related to the actual referents rather than as properties of mental representations.

One of the major pieces of evidence supporting the grounded view comes from the so-called concreteness effect, a well-known behavioural advantage in processing perceptually-based concrete concepts compared to abstract concepts (e.g., De Groot, 1989; Fliessbach et al., 2006; Kroll & Merves, 1986; Schwanenflugel & Stowe, 1989). Within a grounded cognition framework, this effect is indeed attributed to the fact that referents of concrete words are more related to direct experience. Under these conditions, the automatic re-activation of sensorimotor experience associated with perceptually-related concrete referents would facilitate access to their meaning (Connell & Lynott, 2012; Paivio, 1990).

However, recent evidence suggests that the concreteness effect is not necessarily related to direct experience. Indeed, a study by Bottini et al. (2021) found that the advantage in processing concrete concepts holds even for concrete visual words (e.g., *rainbow*) in blind individuals who clearly cannot rely on embodied visual experiences. Words considered to be concrete are processed faster, regardless of the availability of direct visual experience with their referents. Thus, the concreteness effect seems to be dissociated from the degree of perceptual experience we can have with concepts. This result scales down the weight attributed to direct experience from grounded language and casts doubts on the validity of the assumption that perceptual judgements represent purely embodied properties of the objects that are acquired through direct sensory experience.

To further investigate this issue, we tested how accurate visual intuitions about objects are when direct visual experience is missing. Specifically, we tested whether perceptual judgments in blind individuals – and as a control in sighted – align with objective data about the visual world (i.e., diverge from their own experience for which direct visual experience is missing). As an objective basis for evaluating the accuracy of perceptual intuitions about the external world, we considered Image-based Frequency (IF), here adopted as a ground truth measure approximating whether and how much an object is available in the visual environment (Petilli et al., 2022). Thus, using such a data-driven approximation of the visual world allowed us to evaluate the relationship between subjective intuitions (ratings) and the objective status of things in the visual environment. This relationship was also examined taking into account the effects driven by other sensory modalities, to control for their potential influence in signalling object availability.

Given these premises, a radical grounded perspective can anticipate one unique scenario: perceptual intuitions of sighted but not blind individuals are predicted by IF. Indeed, in sighted individuals, this construct approximates properties of concepts that are directly graspable through visual experience and is thus expected to be related to both concreteness (concrete concepts are more visually experienceable than abstract concepts) and imageability (visually experienceable objects can also be easier to mentally imagine). However, following the grounded framework, one would expect such an effect to disappear in blind individuals since they cannot experience word referents visually. Otherwise, the intuitions of blind people would align with objective data about the visual world. This potential scenario would suggest that visual experience is not a prerequisite for developing intuitions on the availability of things in the external visual environment and using these intuitions to inform concreteness/imageability judgments.

## Methods

### Image-based Frequency

**Figure 1:**
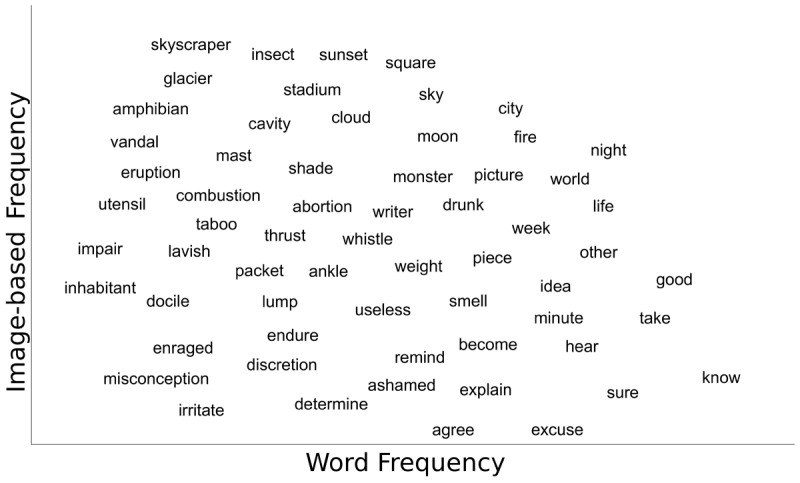
Scatter plot showing for sample words the distribution of IF as a function of word frequency.

IF is computed from Flickr frequency US (Petilli et al., 2022), a measure extracted from the social media Flickr (www.flickr.com) an online platform where people share their photographs online and then assign tags enabling searching for pictures that match those tags. In its original format, Flickr frequency US is operationalised as the Zipf transformed number of images tagged with a given word label uploaded in the US on Flickr. At a surface level, Flickr frequency collects the words that come to the mind of the taggers while seeing an image. When this behaviour is repeated across millions of people and billions of images, it ends up representing a collection of word forms elicited by visualisable entities. However, in this original format, this metric still captures a hybrid construct lying at the intersection between vision and language. Specifically, on the one hand, Flickr frequency captures the availability of the word referent in the (real and online) visual environment and, on the other hand, the frequency of the labels used to define it. As indicated by Petilli et al. (2022), taking the residuals of the regression with Flickr frequency as the dependent variable and lexical frequency (here Laplace transformed CELEX frequency from Baayen et al., 1996) as a predictor produces a metric capturing the portion of variance uniquely captured by the Flickr measures, once the information encoded in word occurrences is accounted for (see supplementary material S1 for a different procedure using Varimax PCA as an alternative method). Here we called this measure IF and used it as a data-driven ground truth that estimates the extent to which something is actually visually present in (a proxy of) the world. As can be seen from Figure 1, after partialling out the contribution of word frequency, we end up with a data-driven measure of the word referents that is independent of word usage: words whose referents are visually experienceable tend to be distributed in the upper part of the scale and words whose referents are hardly available in the visual environment in the lower part. Thus, this transformation ensures that the obtained measure genuinely captures the visual dimension without being influenced by the linguistic availability of the label used for tagging.

### Human Judgments

As perceptual judgements, we extracted ratings from the word norms for blind and sighted by Kerr & Johnson (1991). This database consists of ratings and word associates for 161 nouns obtained from a sample of twelve sighted individuals and twelve early-blind individuals who lost sight completely at birth or very early in life (<2 years old). As perceptual ratings, we considered the measures of imageability and concreteness (detailed instructions given to participants can be found in Toglia & Battig, 1978). In addition to these two variables of interest, familiarity rating was also analysed as a control experience-based variable. The final database of this study consisted of ratings for 158 words resulting from the combination of words from the norms by Kerr & Johnson (1991) and the other two norms used for this study (i.e., CELEX frequency, Baayen et al., 1996; Flickr frequency, Petilli et al., 2022).

### Statistical analysis

Analyses were performed in the R environment (R Core Team, 2021) using linear mixed-effects models (Baayen et al., 2008). In the main analyses ratings of Imageability, Concreteness and Familiarity were separately submitted to a linear mixed-effects regression. IF, Group (i.e. Sighted vs Blind), and the interaction between IF and Group were entered in each model as fixed predictors. In addition, as a control variable, Word frequency (WF; from the CELEX database; Baayen et al. 1996) and its interaction with Group were added as fixed predictors in each model. Concerning the random structure, a by-word random intercept was included. After fitting the models, overly influential outliers were removed via model criticism (2.5 SD of standardised residuals). With this procedure, we eliminated 2.2% of the items in the familiarity analysis, 0.9% in the concreteness analysis, and 1.9% in the imageability analysis.

## Results

A first analysis on word associates showed no significant difference in IF of the strongest associated words produced by blind and sighted individuals (F (1,286) = 0.127, p = .722).^2^ This indicates that the lack of visual experience does not hinder access to highly visual word referents.

**Figure 2:**
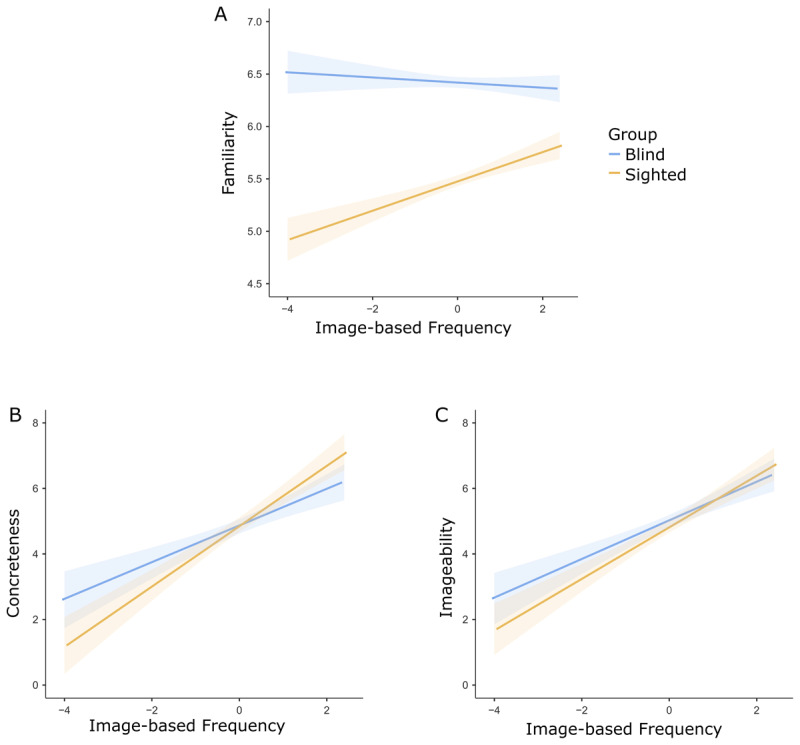
Effect of IF on ratings of familiarity **(A)**, imageability **(C)**, and concreteness **(B)** in blind and sighted individuals.

Concerning rating analyses, a first sanity check was made via the effect of IF on familiarity. The analysis revealed significant interactions between *IF* and *group* (t (148.56) = 6.224, p < .001). The simple effect of *IF* was significant only in the sighted group (t (254) = 5.574, p < .001) in a way that the more a concept can be visualised, the more familiar the concept is judged. On the opposite, *IF* did not significantly predict familiarity in blind individuals (t (254) = -0.97, p = .331) (Figure 2, A). These results indicate that the perceived familiarity with concepts is predicted by how likely it is to visually experience that concept only when such experience is, in principle, possible. Blind individuals rate familiarity independently of the degree of IF of the proposed items, further supporting the validity of the adopted measure.

A different pattern of results emerged for the effects of *IF* on perceptual judgments: a significant interaction between *IF* and *group* was found to predict both *imageability* (t (151.58) = 6.228, p < .001) and *concreteness* (t (153.21) = 3.621, p < .001). The simple effects of *IF* predicting *concreteness* and *imageability* were significant in both blind (imageability analysis: t (178) = 5.266, p < .001; concreteness analysis: t (181) = 6.121, p < .001) and sighted individuals (imageability analyses: t (177) = 8.683, p < .001. Concreteness analyses: t (180) = 8.175, p < .001). Notably these effects hold in blind individuals also when analysing only visually dominant words (imageability analysis: t (105) = 5.332, p < .001; concreteness analysis: t (92) = 5.828, p < .001) and controlling for the perceptual strength in non-visual modalities (imageability analysis: t (179) = 3.712, p < .001; concreteness analysis: t (185) = 4.688, p < .001).

These results indicate that the more a concept is available in the visual environment, the more imageable and concrete the concept is judged in both groups (Figures 2B-2C), although such effect is more evident in the group of sighted individuals. Thus, even when direct visual experience with word referents is absent, the availability of their visual representation in the outside world predicts concreteness and imageability intuitions.

Concerning the effect of WF on familiarity, a significant interaction between *WF* and *group* (t (149.54) = 7.841, p < .001) was also observed. Here, the effect of WF was significant in both groups (blind: t (256) = 4.437, p < .001; sighted: T (255) = 12.734, p < .001), although it was larger in sighted individuals. This indicates that the greater the WF is, the higher the familiarity with the concept. Concerning the effect of *WF* on perceptual judgments, neither main effects of *WF* nor related interactions between *WF* and *group* were found (all *p*s > .103). Unlike IF, WF does not appear to predict perceptual judgments significantly.

## Discussion

The present results provide clear insights into the role of direct experience on human perceptual judgments. IF of word referents predicts perceptual intuitions in both sighted and blind individuals, although to a greater extent in the former. It is worth noting that Kerr & Johnson (1991) already found concreteness and imageability to be highly correlated in blind and sighted individuals, revealing a significant overlap in judgments of the two groups. However, our results paint a more complex picture: first, they provide evidence that people’s perceptual judgements about word referents are related to objective visual aspects of such referents (here, the frequency of their referent in the visible world). Second, they show that such a connection between visual intuitions and the visualisable world is more pronounced in sighted people. This provides evidence that direct visual experience with objects has a part in modelling our perceptual intuitions (for discussion, see Matheson & Barsalou, 2018). At the same time, our results scale down the role of direct visual experience, as attributed by radical view of grounded cognition, by showing that this connection is also found in the intuitions of blind individuals. Taken together, the role of direct visual experience is shown to be important but not critical: even when the direct visual experience of objects is missing, perceptual dimensions of objects can still be estimated accurately (i.e., the more a word has visualisable referents in the outside world, the more concrete and imageable it is judged).

While our study focuses primarily on visual experiences, it is crucial to consider that visual experience is related to the experience of other perceptual modalities (e.g., concepts that can be touched can also be seen, e.g., Vergallito et al., 2020; Lynott et al., 2020). Thus, even when visual experience is absent, other perceptual experiences can inform the availability of objects in the external world. Notably, control analyses have shown that the pattern of results holds even when perceptual strength in non-visual modalities is taken into account, suggesting that such vicarious experiences cannot fully explain our results.

Our findings cast doubts on the validity of the assumption of perceptual judgements representing purely embodied properties of the objects and being a reliable proxy of direct experience. Instead, they fit more closely with the idea that perceptual judgments are outcomes of introspection/abstraction tasks assessing high-level conceptual knowledge that is not necessarily acquired via subjective experience. Taking the semiotic triangle model as a reference (Cherry, 1957; Ogden & Richards, 1923), perceptual intuitions would capture properties of the concept representation (i.e., knowledge, interpretations) instead of properties of the referent (i.e., real-world thing phenomenon).

The opposite considerations can be made for the scale of familiarity with concepts. In that case, the effect of WF on familiarity emerged in both groups, while the effect of IF emerged in sighted individuals only.^3^ This indicates that familiarity with objects is conditioned on the presence of direct experience, be it linguistic (i.e., the more a word is frequent in our linguistic experience, the more familiar its referent is judged) or perceptual experience (i.e., the more a word has visualisable referents in the outside world, the more familiar the object is judged). Considering again the semiotic triangle, familiarity judgments seem to be related to actual experiences and less impacted by abstraction processes.

Our results align with previous literature showing that, despite drastically different perceptual experiences, blind people acquire rich and accurate knowledge of the appearance of visualisable entities (such as animals or colours), which is substantially in agreement with that of sighted people (Bedny et al., 2019; Kim et al., 2019; Marmor, 1978; Shepard & Cooper, 2017; Saysani et al., 2021). These studies suggest that lacking visual experience does not prevent the formation of typical perceptual knowledge, hence explaining how blind people can provide concreteness and imageability judgments that are informed by the perceptual availability of objects.

These findings complement previous studies that question a critical role of direct sensory experience in modelling concreteness of concept representations. Bottini et al. (2021) demonstrated it at an implicit level: the behaviour facilitation in processing concrete words holds even when direct perceptual experience with their referents is missing. Here, we demonstrate it at an explicit level. Objects’ perceptual judgements are accurately produced even without direct perceptual experience with them. Taken together, these results indicate that direct experience is not the only one that can make a concept to be perceived as concrete and imageable.

At this point, the question remains: How intuitions of blind people, who clearly have no experience of the visual world, are nevertheless related by how frequently a human’s eye would gaze on a ball, or a bicycle, or a table? In other words, what else makes a concept concrete and imageable? Sensory experience is just one of the possible sources that inform concept representations about their perceptual properties. In recent studies, a critical role has also been attributed to language in providing all the information required to establish a link to visual experience and extrapolate “visual” knowledge for concepts we have not directly experienced (Campbell & Bergelson, 2022; Günther et al., 2020; Lewis et al., 2019). As also recognised by Wingfield & Connell (2022), “*sensorimotor grounding of word meaning can occur not only via direct, first-hand experience but also indirectly via vicarious experience or inference from linguistic associations*”.

One may question how, in our work, language can be a potential candidate in informing visual knowledge if the influence of WF on IF has been partialled out. In this regard, it is crucial to clarify that the way language conveys semantic information extends beyond the mere frequency of words, which primarily reflects how familiar we are with their label (e.g., Brysbaert & Cortese, 2010). As indicated by Bottini et al. (2021), there are various psycholinguistic variables – even variables not merely appealing to the knowledge of what is physical vs non-physical – which are associated with concreteness and may be “partly dependent on the perceptual origin of concepts”. Among them, there are semantic aspects such as contextual availability (i.e., concrete words are easier to contextualise; Schwanenflugel et al., 1988) and age of acquisition (i.e., concrete words are learned earlier in life; Brown & Watson, 1987), emotional aspects related to arousal (i.e., abstract words tend to have higher emotional arousal; Vigliocco et al., 2009), and formal aspects related to the structure of words (e.g., abstract words are typically longer and tend to be morphologically complex; Reilly & Kean, 2007). Therefore, even excluding linguistic information about WF from IF, several variables could, in principle, make language a viable candidate for conveying perceptual knowledge about the concreteness of objects.

In addition, distributional semantic models have shown that the ability of language to convey semantic information is not limited to the properties of individual words taken in isolation, being subtly related to structural (statistical) relationships between words and their linguistic contexts (e.g., Baroni & Lenci, 2010; see also Günther et al., 2019). “Car” and “vehicle” are semantically similar not because they frequently appear in language but because the two words frequently co-occur with the same other terms. Starting from the distributional statistics of words, these models demonstrate that it is possible not only to extract the semantics of a word based on contextual information (i.e., words with similar meanings tend to appear in similar linguistic contexts) but also to generate concept representations through analogical reasoning. This allows inferences about the semantics of novel concepts (for in-depth discussions in this respect, see Gentner & Asmuth, 2017; Lupyan & Lewis, 2017; Yee, 2019; Günther et al., 2019), including their perceptual aspects. One of the major pieces of evidence comes from a study by Hollis & Westbury (2016). They applied principal component analysis to skip-gram vectors (Mikolov et al., 2013) and found that the second extracted component has face validity as a dimension of concreteness, thus suggesting that concreteness information is encoded in the distributional statistics of words. Likewise, van Paridon et al. (2021) demonstrate that colour-adjective associations, as represented in linguistic distributions, are predictive of colour-adjective associations ratings collected from both blind and sighted individuals, thus providing evidence for language distributions as a potential source of visual knowledge (Lewis et al., 2019). Also, Günther et al. (2020) found that vision-based representations can be predicted from text-based distributional vectors, proposing a viable route for non-experienced referents to be grounded in perceptual experience. Similarly, Louwerse (2011) argues that embodiment findings typically attributed to perceptual simulations can be explained by distributional linguistic information. This is because language usage is intertwined with the physical world, being language often used to communicate about it. This produces statistical redundancies between the structure of the perceivable world and language so that relations between words tend to reproduce the relations between their referents in the real world (Günther et al., 2019; Günther et al., 2020; Johns & Jones, 2012). All of these considerations make clear that language is a plausible candidate for allowing experience by proxy of what people perceive in the outside world.

Our results call into question the traditional interpretation (and application) of concreteness and imageability ratings. Previous studies (e.g., Connell & Lynott, 2012) already criticised these measures for ignoring and distorting the role of particular modalities (Connell & Lynott, 2012; Speed & Brybaert, 2022; Vergallito et al., 2020). However, here we highlight how the issue might be more substantial and related to the non-perceptual nature of these judgments: these ratings seem not to be uniquely and necessarily based on the actual experiences of the rater. These results could explain why other types of perceptual ratings, such as modality-specific measures of perceptual strength, tend to outperform concreteness and imageability in predicting lexical processing (Connell & Lynott, 2012; Speed & Brybaert, 2022). In this sense, one may speculate that, unlike concreteness and imageability, modality-specific ratings might be better at prompting judgements that are really based on actual people’s experiences. However, further study is required to test such a hypothesis.

From a broader perspective, these findings solicit a reconsideration of how we use rating measures in the psychological disciplines. We typically take them as the *explanans* of other human behaviours. However, they are themselves psychological behaviours that need to be explained and understood before being used as the gold standard for explaining other behaviours. Note that this practice may be problematic on the epistemological ground. As pointed out by Jones et al. (2015), it leads to the loophole of predicting behavioural responses (e.g., lexical processing time) from other behavioural responses (i.e., ratings) (see also Günther et al., 2022; Hollis & Westbury, 2016; Petilli et al., 2021). This is particularly problematic when these data are used as independent variables that are supposed to accurately measure the object properties under investigation (e.g., the amount of perceptual interaction we can have with objects). This makes clear the need for data-driven measures that more closely approximate the actual experience human have at the input level in order to be used as independent predictors of human behaviours.

To conclude, it is worth mentioning that the dataset by Kerr & Johnson (1991), on which our analyses are based, is rather modest compared to current research standards. Despite this limitation, a resource like this nevertheless provides insightful and worthwhile data from a special population that presents considerable difficulties for data collecting. However, replicating these results on a larger sample of words (and other participants) would be important to consider the evidence here as fully established.

## Data Accessibility Statement

The dataset and the script for the analyses reported here are openly available on the Open Science Framework (https://osf.io/qxetr/).

## Footnotes

Especially for the construct of imageability, for which it is pointed out that people tend to rely on visual experience when generating ratings (Connell & Lynott, 2012).

Note that word associates produced by blind and sighted individuals do not significanlty differ in terms of dominant perceptual modality (perceptual norms from Lynott et al., 2020) (X^2^(5) = 2.048, p = .84; see Supplementary Materials S0).

Caution is required in interpreting results on familiarity in blind individuals because of the low variability in these ratings which might have limited statistically significant effects to emerge. However, the effect WF on familiarity grant us cautious optimism about the validity of these analyses as it suggests that there is sufficient variability to observe potential effects on familiarity when clearly present.
